# Supplementary material for: Isolation and Characterization of Paenibacillus polymyxa B7 and Inhibition of Aspergillus tubingensis A1 by Its Antifungal Substances
Source: Int J Mol Sci. 2024 Feb 12;25(4):2195. doi: 10.3390/ijms25042195 (PMC10889487; doi:10.3390/ijms25042195)
Supplement: Supplementary file 1 [file ijms-25-02195-s001.zip › ijms-2840452-supplementary.pdf]

Supplementary Figure

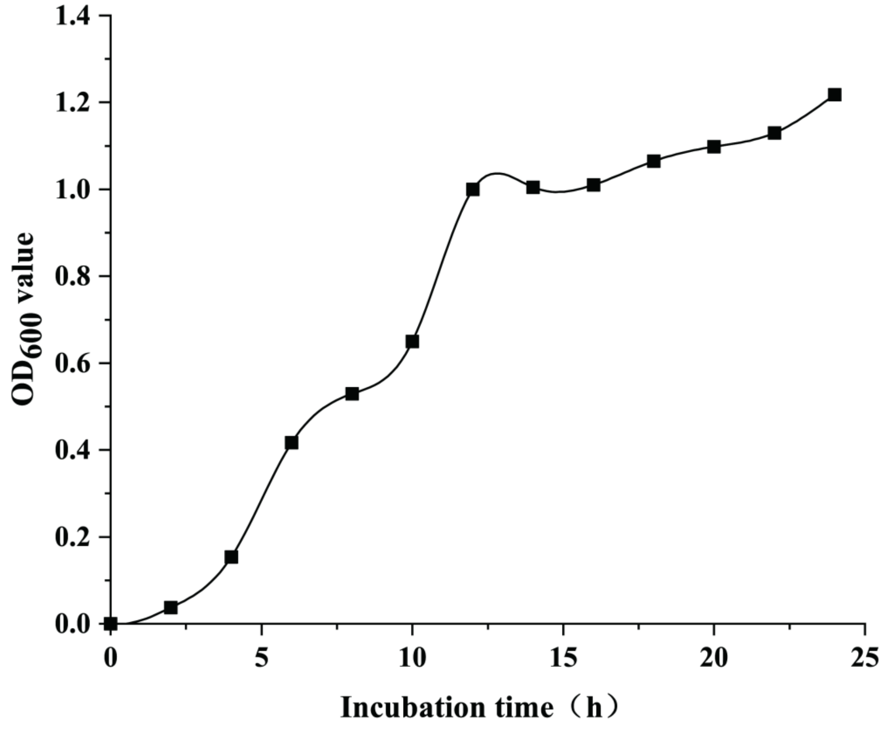

Figure S1. Growth curve of antagonistic bacteria. Each tube with 10 ml of LB liquid medium, were incubated for 24 hours with 1% inoculum and cultivated under the shaker. Samples were taken every two hours during the 26-hour incubation period and placed in the refrigerator. Finally, the samples were diluted and the absorbance values at OD_{600} were determined uniformly. The logarithmic growth phase began at 4 h, and the number of *Bacillus* cells grew exponentially until 14 h.

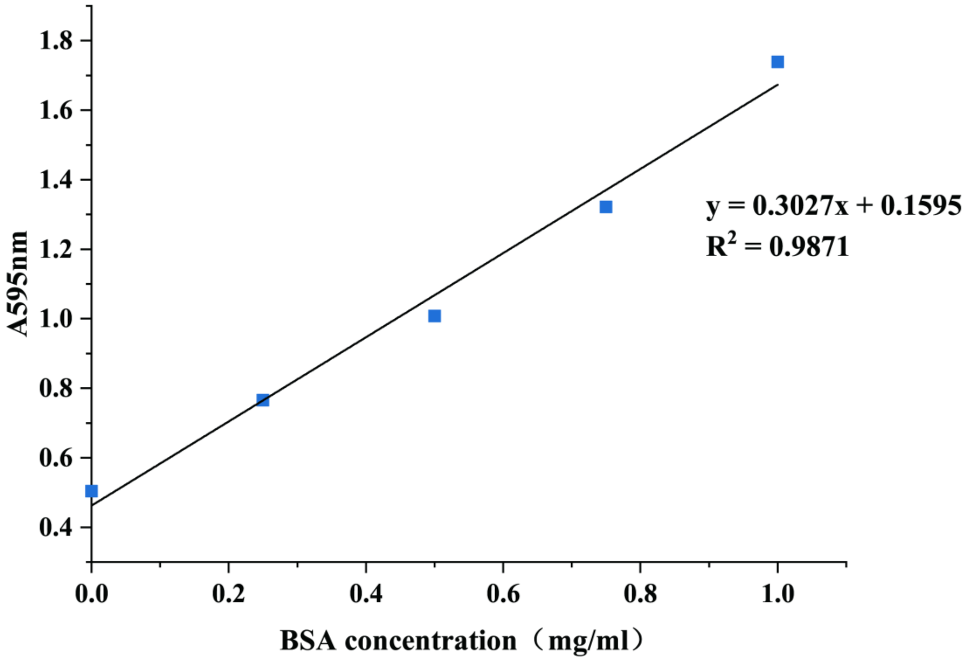

Figure S2. Protein Standard Curve. According to the Bradford Protein Assay Kit, protein standards were configured by dissolving the samples through PBS buffer. Firstly, 5 μl of protein standards of different concentrations were taken and were added to the protein standard wells of the 96-well plate. 5μl of samples were taken into other sample wells of the 96-well plate. If the sample was less than 5 μl, standard diluent was added to make up to 5 μl. Secondly, 250 μl of G250 Staining Solution was added to each well. At last, A595 was determined using an enzyme marker, and then the standard curve and the sample volume were used to calculate the protein concentration.

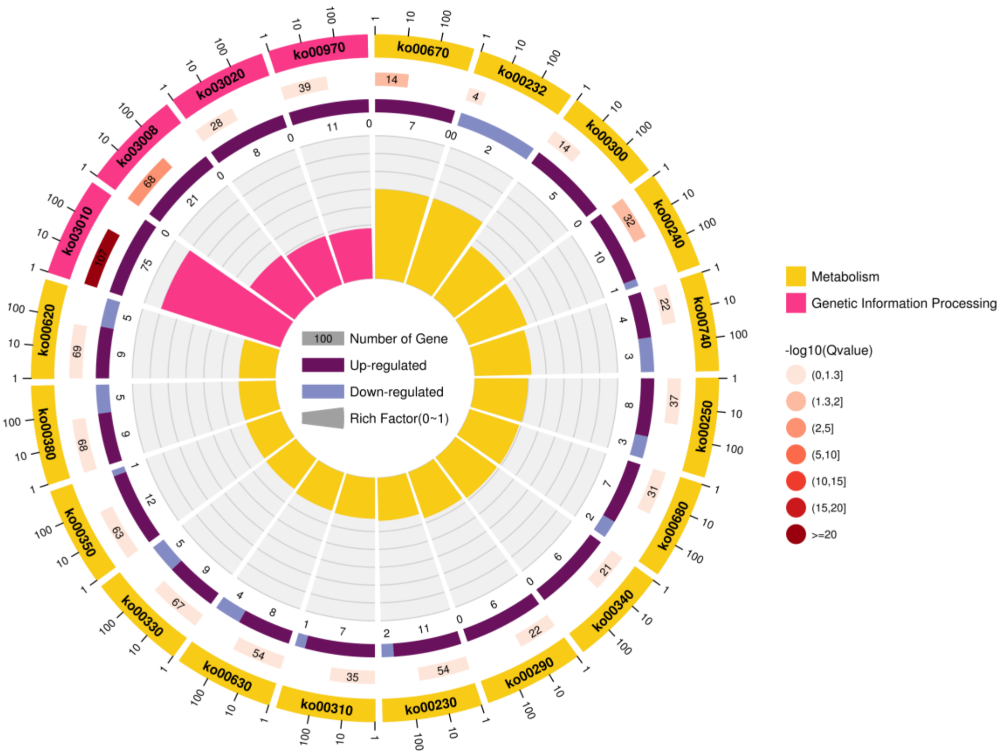

Figure S3. KEGG enrichment circle diagram: (First circle: top 20 enriched pathways, outside the circle is the sitting scale for the number of differential genes. Different colors represent different A classes; Second circle: the number of the pathway in the differential gene background and the Q value. The longer the number of differential genes in the background, the longer the bar is, the smaller the Q value is, the redder the color is; Third circle: up-down differential gene ratio bar, dark purple represents the up-down differential gene ratio, light purple represents the down-down differential gene ratio; specific values are shown at the bottom; Fourth circle: RichFactor value of each pathway (the number of differential genes in the pathway divided by the number of differential genes in the pathway), the background grid value (the number of differential genes in the background grid), the number of differential genes in the background grid, the number of differential genes in the background grid. all quantities), background grid lines, each cell represents 0.1)
